# Linking of Rasch-Scaled Tests: Consequences of Limited Item Pools and Model Misfit

**DOI:** 10.3389/fpsyg.2021.633896

**Published:** 2021-07-06

**Authors:** Luise Fischer, Theresa Rohm, Claus H. Carstensen, Timo Gnambs

**Affiliations:** ^1^Leibniz Institute for Educational Trajectories, Bamberg, Germany; ^2^Psychological Methods of Educational Research, University of Bamberg, Bamberg, Germany

**Keywords:** Rasch model, item response theory, linking methods, model misfit, anchor- items design, limited item pools

## Abstract

In the context of item response theory (IRT), linking the scales of two measurement points is a prerequisite to examine a change in competence over time. In educational large-scale assessments, non-identical test forms sharing a number of anchor-items are frequently scaled and linked using two− or three-parametric item response models. However, if item pools are limited and/or sample sizes are small to medium, the sparser Rasch model is a suitable alternative regarding the precision of parameter estimation. As the Rasch model implies stricter assumptions about the response process, a violation of these assumptions may manifest as model misfit in form of item discrimination parameters empirically deviating from their fixed value of one. The present simulation study investigated the performance of four IRT linking methods—fixed parameter calibration, mean/mean linking, weighted mean/mean linking, and concurrent calibration—applied to Rasch-scaled data with a small item pool. Moreover, the number of anchor items required in the absence/presence of moderate model misfit was investigated in small to medium sample sizes. Effects on the link outcome were operationalized as bias, relative bias, and root mean square error of the estimated sample mean and variance of the latent variable. In the light of this limited context, concurrent calibration had substantial convergence issues, while the other methods resulted in an overall satisfying and similar parameter recovery—even in the presence of moderate model misfit. Our findings suggest that in case of model misfit, the share of anchor items should exceed 20% as is currently proposed in the literature. Future studies should further investigate the effects of anchor item composition regarding unbalanced model misfit.

## Introduction

Investigating differences between groups that were administered non-identical test forms in an item response theory (IRT) framework requires aligning two (or more) test forms onto a common scale, which is known as linking (Kolen and Brennan, 2014). As the process of linking requires an overlap of information among scales, this is frequently achieved by using an anchor-items design (Vale, 1986, p. 333–344), where test forms share a number of common items. Linking is a common procedure in the context of large-scale assessments (LSA) in educational measurement such as the *Programme of International Student Assessment* (PISA) or the *American National Assessment of Educational Progress* (NAEP), which are characterized by large item pools and sample sizes. As such, LSAs provide an appropriate field for the application of 2-parameter logistic (2PL) and 3-parameter logistic (3PL) models (Birnbaum, 1968, p. 397–472) as a basis for scaling and linking the data. In contrast, in contexts which are characterized by a limited pool of items and small to medium sample sizes (as often is the case in studies with restricted economical resources or longitudinal designs) the sparser Rasch (1960) model is a suitable alternative (Sinharay and Haberman, 2014, p. 23–35). As of yet, the linking of Rasch-scaled data in this specific context was rarely researched.

In this article, we systematically investigate the linking of Rasch-scaled data based on limited item pools and small to medium sample sizes. To mimic applied settings, the data simulation mirrored a longitudinal design similar to the German *National Educational Panel Study* (NEPS; Blossfeld et al., 2011). Although mean change in a longitudinal design is often larger than differences among groups in a cross-sectional design, the linking is conceptually equivalent (von Davier et al., 2006). More specifically, the present simulation study deals with the issues of comparing and evaluating the performance of four IRT linking methods and investigating the absolute and relative number of anchor items required in these contexts. Moreover, as strict assumptions are made on equal item slopes in the Rasch model that are hardly met in empirical data, the robustness of linking methods toward model-data misfit is investigated.

In the following sections, we describe the Rasch model, the four common IRT linking methods, as well as challenges inherent to linking with limited item pools and sample sizes. Next, we describe the set-up of the simulation study and report the present findings. Finally, we discuss implications and limitations of our results.

## The Rasch Model

In the Rasch (1960) model, it is assumed that the probability *P* of person *n* ∈ 1…*N* to correctly answer a dichotomous item *i* ∈ 1…*I* is conditioned on the interaction of two parameters, that is, a person’s ability β_*n*_ and an item’s difficulty δ_*i*_ on a latent continuum:

(1) $$P\left(X_{n\,i}=1|\beta_{n},\delta_{i}\right)=\frac{\exp\left(\beta_{n}-\delta_{i}\right)}{1+\exp\left(\beta_{n}-\delta_{i}\right)}.$$

Compared to 2PL and 3PL models, no parameter for item discrimination α*_*i*_* is directly incorporated. Therefore, a higher precision in (anchor) item difficulties can be obtained at smaller sample sizes (Thissen and Wainer, 1982, p. 397–412) in the Rasch (1960) model.

Every item *i*, belonging to a test form fitting a Rasch model, measures the same latent construct with equal item discriminations *α_*i*_* at all levels of β. Stated differently, items are not allowed to differ in their power to discriminate among persons (Wright, 1977, p. 97–116) and, thus, an irrevocable rank order among individuals β_1_ … < β*_*n*_* < … β*_*N*_* is determined based on the sufficient statistics of the person sum scores. As it can be challenging for empirical data to fully meet this strict specification, the question is *not* whether the data does or does not fit to a model, but is rather a “matter of degree” (Meijer and Tendeiro, 2015). As the weighting by α*_*i*_* of person sum scores is ignored in case of Rasch model-data misfit (i.e., α*_*i*_* ≠ 1), sample mean and variance estimates of the latent variable might be biased (Humphry, 2018, 216–228) as they are based on (1). Additionally, the precision of (anchor) item difficulties decreases (Thissen and Wainer, 1982, p. 397–412).

## IRT Linking Methods

In IRT, only individual proficiencies and item difficulties located on equally defined scales are directly comparable over different measurement occasions (Kolen and Brennan, 2014). As such, prior to investigating proficiency development or group differences in an IRT framework, it is required to align two (or more) test forms onto a common scale (e.g., using an anchor-items design). As anchor item parameters are assumed to be measurement invariant and, thus, to maintain their difficulty over time, they allow for displaying an individual’s change in proficiency. Several IRT linking methods exist, differentially “translating” the linking information during the linking process. The present study focuses on IRT linking methods compatible with Rasch-type models (van der Linden and Hambleton, 2013) that preserve uniform item discrimination parameters across the linked scales (Fischer et al., 2019, p. 37–64). The different linking methods scale the different test forms either separately or concurrently. In separate calibration methods, anchor item difficulty parameters of each test form are estimated prior to the linking process. This subsequently extracted link information is then implemented uniquely by each linking method. Hence, a once established reference scale remains unchanged throughout the course of measurement. In the present section, the three different calibration methods (1) fixed parameter calibration (Kim, 2006, p. 355–381), (2) mean/mean linking (Loyd and Hoover, 1980, p. 179–193), and (3) weighted mean/mean linking (van der Linden and Barrett, 2016, p. 650–673) are shortly described. Additionally, (4) a one-step approach of simultaneously calibrating and concurrently linking all test forms (e.g., Kim and Cohen, 1998, p. 131–143) is presented.

### Fixed Parameter Calibration (FPC)

The parameter of anchor item *l* ∈ 1*L* with *L*⊆*I* of test form *A* intended to link are fixed using the estimated item parameters of the referencing test form *B*:

(2) $$\delta_{A\,l}=\delta_{B\,l},$$

leaving no possibility for differences in anchor item parameters. Test forms based on a longitudinal design that vary in their sets of anchor items are linked sequentially (i.e., after test form t_2_ is linked to t_1_, t_3_ is linked to t_2_ and so on).

### Mean/Mean Linking (m/m)

To link test form *A* to test form *B* and, therefore, obtain the linked item difficulty parameters δ^∗^_*Ai*_, the linking constant *v* is added to each item δ_*Ai*_:

(3) $$\delta_{A\,i}^{*}=\delta_{A\,i}+v;$$

with *v* being the difference of the means of the *anchor item* difficulty parameters δ_*AL*_ and δ_*BL*_:

(4) $$v=M\,\left(\delta_{B\,L}\right)-M\,\left(\delta_{A\,L}\right).$$

After the linking results that M(δ^∗^_*AL*_) = M(δ_*BL*_).

### Weighted Mean/Mean Linking (wm/m)

This approach incorporates estimation precision in weighting the anchor item difficulty parameter estimates by the inverse of their squared standard errors, $S\,E_{\delta_{A\,l}}^{-2}$ and $S\,E_{\delta_{B\,l}}^{-2}$, prior to conducting a mean/mean linking, replacing *v* with

(5) $$v^{\prime }=\frac{\left(\sum_{l=1}^{L}\delta_{B\,l}\,S\,E_{\delta_{B\,l}}^{-2}\right)}{\left(\sum_{l=1}^{L}S\,E_{\delta_{B\,l}}^{-2}\right)}-\frac{\left(\sum_{l=1}^{L}\delta_{A\,l}\,S\,E_{\delta_{A\,l}}^{-2}\right)}{\left(\sum_{l=1}^{L}S\,E_{\delta_{A\,l}}^{-2}\right)}.$$

As such, the precision of the anchor item difficulty estimates of test forms *A* and *B* is taken into account, aiming at reducing the link error (i.e., a reflection of the uncertainty introduced to the link due to the selection of link items). In other words, *v’* is identical to *v* when the anchor item difficulty parameter estimates have equal standard errors within a test form. Hence, weighted mean/mean linking is expected to outperform mean/mean linking when anchor items differ in precision.

### Concurrent Calibration (CC)

All test forms are scaled concurrently in a one-step estimation procedure, constraining the anchor item difficulties across time points. As such, anchor item difficulties are simultaneously fitted to best meet the characteristics of all measurement points interacting with the samples’ proficiency distributions.

Imprecision of (anchor) item difficulty estimates is reflected in their increased standard error (*SE*). In order to minimize estimation imprecision in item and person parameter estimates at *each time point*, a sample’s proficiency and a test’s difficulty should considerably overlap (i.e., also known as test targeting). In other words, the mean and variance of some test items’ difficulty should closely fit the proficiency distribution of a respective sample. Of course, this claim is also true for sets of anchor items. Since sets of anchor items are administered repeatedly, they are expected to fit *several* proficiency distributions simultaneously. Consequently, the more diverging these proficiency distributions are, the more wide-spread a section of the latent scale needs to be covered by the sets of anchor items. It is to be noted that anchor items located at the outer edges of these joint ability distributions are prone to an increased *SE*. Svetina et al. (2013, p. 335–360) reported that a mismatch between item and person parameter distributions (i.e., if the item difficulties are, on average, too easy or too difficult as compared to the average proficiency distribution of the sample) impacted the recovery of item difficulty parameters more than the person parameter estimates. As such, linking methods that do not derive the linking information from the item level may be more “forgiving” with respect to imprecise estimates, as they are more likely to cancel out. As was shown by van der Linden and Barrett (2016, 650–673), the linking result of wm/m was superior to m/m in situations when anchor items did not perfectly display the samples’ ability distribution. Therefore, the estimated amount of change is expected to be closer to its true value, compared to a result that is based on linking methods that link on the item level. Consequently, the method of weighted mean/mean linking that accounts for possible imprecisions in difficulty estimates by weighting anchor items by their *SE*s is expected to outperform the linking methods mean/mean linking, concurrent calibration and fixed parameter calibration (in the given order).

## Challenges for the Linking of Rasch-Scaled Data

### Model-Data Misfit

There is a rather limited body of research examining the influence of Rasch model-data misfit on linking results. For example, Zhao and Hambleton (2017, p. 484) showed that in an LSA context with large sample sizes (*N* = 50,000) and long tests (78 items) with many anchor items (*k* = 39) fixed parameter calibration was more sensitive to model misfit and more robust against sizable ability shifts (up to 0.5 logits) as compared to linking methods that preserve the relation between item difficulty parameters during linking (i.e., mean/sigma method; Marco, 1977, and the characteristic curve methods; e.g., Stocking and Lord, 1983). As such, model fit was crucial to the appropriate use of FPC. So far, no research investigated the sensitivity and reactivity of IRT linking methods toward model misfit under more realistic conditions with smaller samples and shorter tests. Following Zhao and Hambleton (2017, p. 484), we hypothesized that FPC would be more sensitive toward model misfit as compared to CC, whereas m/m and wm/m would be least affected.

### Number of Anchor Items

Kolen and Brennan (2014) formulated a rule of thumb for large item pools, proposing that the number of anchor items should make up about 20%. Nothing was stated for item pools consisting of less than 200 items. If a single anchor item would fully reflect the latent construct and was free of differential item functioning (DIF), this item would be sufficient for aligning two tests on a common scale. As this hardly is the case in practice, several anchor items are typically used in operational tests. Generally, a larger number of anchor items is assumed to reduce random link error and, thus, is expected to more precisely recover the true value of mean change. Moreover, a larger number of anchor items increase the content validity of the link. However, when test length is rather short (i.e., 25 items) and changes in proficiency between measurement points of a longitudinal sample are expected to be sizable (i.e., ≥0.25 logits; Zhao and Hambleton, 2017, p. 484), one repeatedly administered identical test form (i.e., 100% anchor items) would potentially affect test targeting and test reliability. In other words, when samples differ substantially in their mean proficiencies, the number of anchor items in a short test form becomes a question of measurement precision at each measurement point. More precisely: An item’s difficulty that matches a sample’s mean ability well at the first measurement point *t*_1_ cannot match a sample’s mean ability well at the second measurement point *t*_2_ when there was a significant change in the sample’s ability between *t*_1_ and *t*_2_. Here is a demonstrative example: We assume that there is a significant change in ability of a sample that is administered two test forms with a length of 15 items sharing a number of 10 anchor items. We further assume that these 10 anchor items have a very good test targeting at *t*_1_. From that follows that the test targeting of these 10 anchor items would have to be worse at *t*_2_, affecting test reliability. Furthermore, administering items repeatedly may provoke memory effects that become more probable to emerge with an increasing number of anchor items. This leads to the question which proportion of anchor items can optimally balance measurement precision and linking information. Is the advice of a 20% anchor items share transferable to (rather) short test forms? In addition, questions about the minimum number of anchor items necessary to accurately display growth, and how model-data misfit interacts with the number of anchor items, remain.

To sum up, the present study aimed at comparing the performance of four common IRT linking methods (fixed parameter calibration, mean/mean linking, weighted mean/mean linking and concurrent calibration) based on Rasch-scaled simulated data. Particularly, we examined to what degree the number of anchor items and the degree of Rasch model-data misfit affected the linking for the different approaches.

## Methods

### Data Generation

We simulated data for four time points (t_1_–t_4_) to measure within-individual growth in an anchor-items design (Vale, 1986, p. 333–344). The simulation was modeled after empirical data from the German National Educational Panel Study (NEPS; Blossfeld et al., 2011). The NEPS aims at measuring competence development over the life span. Therefore, respondents from different age cohorts (e.g., 10- or 15 years old) are followed and receive repeated competences tests at different ages in their lives. Thus, the measured competences of these respondents are characterized by large changes across childhood and adolescence. As such, the NEPS is confronted with various methodological issues such as linking test forms administered at different ages that vary significantly in their average difficulty. Nonetheless, these tests were intended to measure the same underlying construct. To gain deeper insight in the linking process under these conditions the setup of the present simulation study was oriented on reading tests, that were administered in grades 5, 7, 9, and 12 of the NEPS (Pohl et al., 2012; Krannich et al., 2017; Scharl et al., 2017). The observed mean proficiencies (in logits) were 0.0, 0.7, 1.2, and 1.5, respectively. Similar, we randomly drew proficiencies from normal distributions with these means and unit variances. We simulated responses to four test forms each including 25 items. The true item difficulties were generated in R 3.5.2 (R Core Team, 2018) from multivariate normal distributions matching the proficiency distributions (see Table 1), thus, resulting in a good test targeting. As the anchor items had to fit two distributions simultaneously (t_1/2_, t_2/3_, t_3/4_), they were set to fall between two distributions (see Tables 1, 2). Anchor items maintained their difficulty parameters over time and as such met the assumption of measurement invariance. The item response models were estimated using the R-package TAM 3.1-26 (Kiefer et al., 2018) that iteratively updated the prior ability distribution using the EM algorithm (Bock and Aitkin, 1981, p. 443–459) during MML estimation (Kang and Petersen, 2012, p. 311–321). Due to the need of extensive computational power for the concurrent calibration, the quasi Monte Carlo estimation algorithm (based on 1,000 nodes) was used, whereas the Gauss-Hermite quadrature was used for the other linking methods. The original code for data generation is provided at https://osf.io/7vta8/.

**TABLE 1:** True item difficulty and item discrimination parameters of the four test forms (t_1_–t_4_).

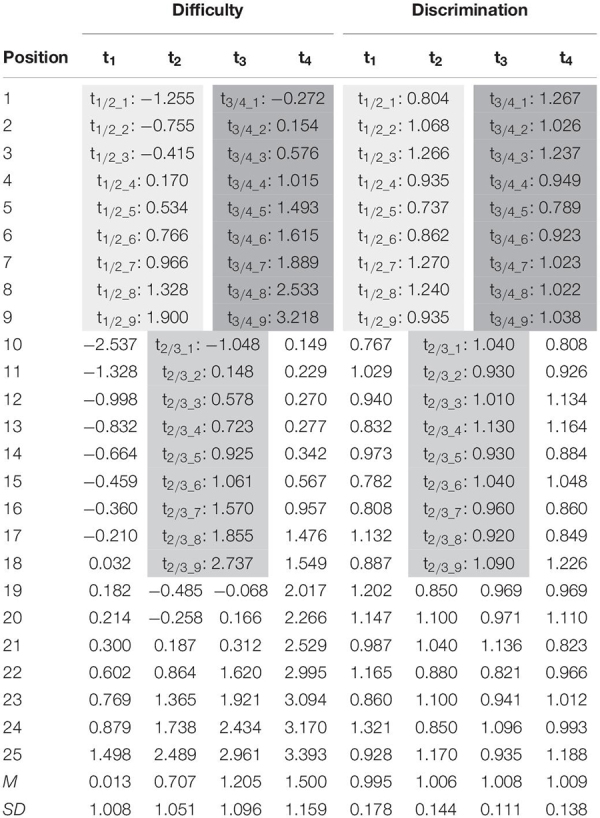

**Framed parameters represent anchor items linking adjacent measurement points. Position = item position in each test form; t_1/2_, t_2/3_, t_3/4_ = true anchor item parameters linking measurement points t_1_, t_2_, t_3_, t_4_; M = mean of 25 true item parameters; SD = standard deviation of 25 true item parameters.**

**TABLE 2:** Descriptive statistics of the true anchor item parameters split by the experimental factor number of anchor items.

	t_1/2_				t_2/3_			t_3/4_		
			
	Anchor item difficulty parameters									
	
Anchor	Position	*M*	*SD*	Position		*M*	*SD*	Position	*M*	*SD*
3	2,5,8	0.369	1.051	2,5,8		0.976	0.855	2,5,8	1.393	1.193
5	2,3,4,6,9	0.333	1.050	1,5,6,7,8		0.873	1.138	2,3,4,6,9	1.316	1.193
7	1,2,4,5,6,7,9	0.332	1.066	1,3,4,5,6,8,9		0.976	1.169	1,3,4,5,6,7,9	1.362	1.096
9	1–9	0.360	1.022	1–9		0.950	1.074	1–9	1.358	1.120

	**Anchor item discrimination parameters**									
	
	**Position**	***M***	***SD***	**Position**		***M***	***SD***	**Position**	***M***	***SD***

3	2,5,8	1.015	0.256	2,5,8		0.927	0.006	2,5,8	0.946	0.136
5	2,3,4,6,9	1.013	0.160	1,5,6,7,8		0.978	0.058	2,3,4,6,9	1.035	0.123
7	1,2,4,5,6,7,9	0.944	0.178	1,3,4,5,6,8,9		1.023	0.077	1,3,4,5,6,7,9	1.032	0.171
9	1–9	1.013	0.206	1–9		1.006	0.076	1–9	1.030	0.148

**Anchor = Number of anchor items used for linking; t1/2, t2/3, t3/4 = true anchor item parameters linking adjacent measurement points; Position = selected anchor items out of anchor set (see Table 1 for anchor item identification); M = mean of true anchor item parameters; SD = standard deviation of true anchor item parameters.**

### Experimental Factors

For each simulated sample the four test forms (t_1_–t_4_) were linked based on the four linking methods of fixed parameter calibration, mean/mean linking, weighted mean/mean linking, and concurrent calibration. Model fit was varied in two ways by either meeting the Rasch model assumptions of constant item discriminations (α*_*i*_* = 1) or modeling slight deviations (see Table 1) by drawing them from *N*(1, 0.14^2^). The resulting item discrimination parameters mirrored empirical results from a 2PL scaling of the tests (Krannich et al., 2017) mentioned above and, thus, were assumed to reflect a moderate degree of misfit within the range of operational proficiency test forms. Linking was based on a number of 3 (12%), 5 (20%), 7 (28%), or 9 (36%) common items among adjacent test forms (see Table 1). While 5 anchor items fell in line with recommendations in the literature (Kolen and Brennan, 2014), the other conditions evaluated the consequence of using more anchor items (7 or 9) or relying on a very restricted set of anchor items. The sample size condition was varied twofold (*N* = 500, *N* = 3,000). Overall, in addition to the within-subject experimental factor (four IRT-linking methods), three between-variable experimental factors—model fit (2), number of anchor items (4) and sample size (2)—were manipulated resulting in 4 × 2 × 4 × 2 = 64 conditions. Each within-subject experimental condition was simulated 100 times, to control for random sampling error.

### Outcome Variables

We examined (a) the convergence rate of models as well as calculated (b) bias, (c) relative bias, and (d) root mean square error (RMSE) for sample mean and variance of the latent variable. The bias was calculated as ${\hat{\tau }}_{d}-\tau$, with ${\hat{\tau }}_{d}$ denoted as parameter estimate of the *k*th replication of condition *d* and τ denoting the true parameter value. The bias was then averaged over all *k* replications of each condition. Serving as an effect size, the relative bias was calculated as a proportion of $\left({\bar{\tau }}_{d}-\tau \right)/\tau ,$with ${\bar{\tau }}_{d}$ being the averaged parameter estimate over all *k* replications. Following Forero et al. (2009, p. 625–641), we considered a relative bias below 10% as acceptable. The RMSE gives the precision of a parameter estimate and was calculated as $\sqrt{\frac{1}{c}\,\sum_{k=1}^{c}{\left({\hat{\tau }}_{k}-\tau \right)}^{2}}$. As such the RMSE was defined as the square root of the mean of the squared bias.

## Results

Only negligible differences among the three linking methods of fixed parameter calibration, mean/mean and weighted mean/mean linking were found with regard to the outcome variables bias, relative bias and RMSE. Results are, therefore, reported combined. Descriptive statistics split by linking methods and experimental factors of the respective outcome variables are reported in Supplementary Tables 2–5.

### Convergence Rates

Only 50.8% (i.e., 813 of 1,600 samples) of the models calibrated concurrently converged. Non-convergence was split about evenly among the experimental factors of sample size and model-data misfit, but varied substantially among different numbers of anchor items (see Supplementary Table 1). Moreover, in-depth analyses (not reported in this manuscript) of successfully converged concurrently calibrated models revealed that smaller numbers of iteration steps did not necessarily lead to a more precise parameter estimation. As these findings were questioning the applicability of concurrent calibration in settings based on small absolute numbers of anchor items, it was excluded from further analyses. In contrast, all models that were calibrated separately (fixed parameter calibration, mean/mean linking and weighted mean/mean linking) converged.

### Sample Mean

#### Bias

**FIGURE 1:**
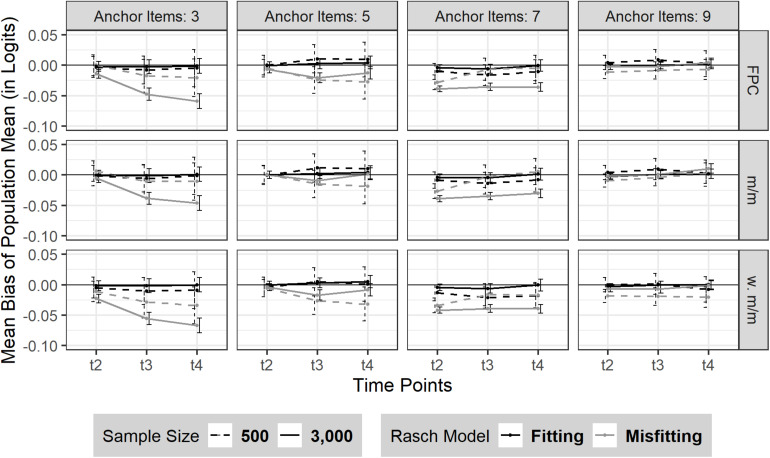
Bias of sample mean over three time points (t_2_–t_4_). The figure is split by three linking methods and the experimental factors number of anchor items, sample size and Rasch model-data fit. FPC = fixed parameter calibration, m/m = mean/mean linking, w. m/m = weighted mean/mean linking. 95% confidence intervals are depicted.

Overall, there was no (change in) bias over the three time points (*M*_*t2*–*t4*_ = 0.00; t_1_ was constrained to 0 due for model identification) in the absence of model misfit. Neither sample size nor the number of anchor items had a substantial effect on the consistency of the bias of sample mean in the absence of model misfit (see Figure 1); although the bias was marginally smaller when sample size was *N* = 3,000 compared to *N* = 500. However, the sample mean was less well recovered in case of moderate model misfit (see Figure 1 and Supplementary Table 2). Rather consistently, the sample mean was underestimated over the three time points, t_2_–t_4_, in all conditions but the conditions based on linking using 9 (36%) anchor items. The amount and pattern of the bias of sample mean emerged in a rather heterogeneous picture among time points and the number of anchor items. Overall, we found that the bias of sample mean rather decreased with an increasing number of anchor items.

#### Relative Bias

The relative bias was always explicitly below 10% and only rose above 5% in 2 conditions (see Supplementary Table 2) and was, thus, considered acceptable.

#### RMSE

**FIGURE 2:**
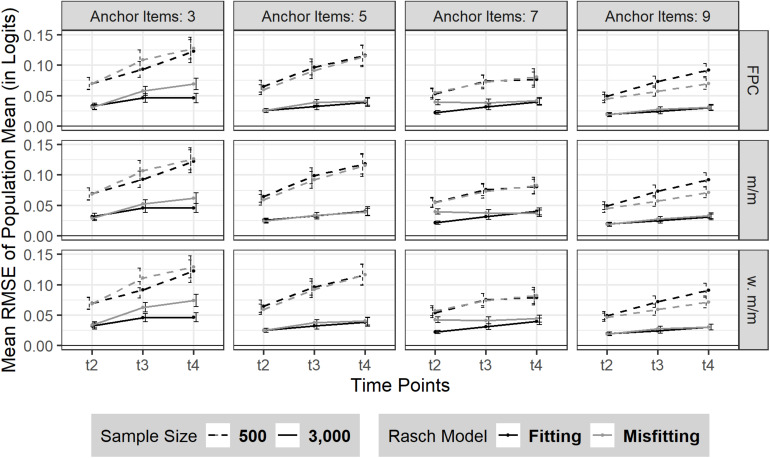
RMSE of sample mean over three time points (t_2_–t_4_). The figure is split by the three linking methods and the experimental factors number of anchor items, sample size and Rasch model-data fit. FPC = fixed parameter calibration, Mean/Mean = mean/mean linking, w. Mean/Mean = weighted Mean/Mean. 95% confidence intervals are depicted.

The RMSE of sample mean linearly increased from t_2_ to t_4_ (see Figure 2). Sample size influenced the amount of RMSE as expected: smaller sample size led to a bigger RMSE with marginally steeper slope over time (*N* = 500: t_2_ = 0.06 (*SD* = 0.04), t_3_ = 0.08 (*SD* = 0.06), t_4_ = 0.10 (*SD* = 0.08) compared to a larger sample size (*N* = 3,000: t_2_ = 0.03 (*SD* = 0.02), t_3_ and t_4_ = 0.04 (*SD*_*t3*,t4_ = 0.03). Additionally, the RMSE of sample mean was in general smaller when linking based on a larger number of anchor items. More precisely, a larger number of anchor items seemed more beneficial for a smaller sample size (*N* = 500). It has to be noted that a moderate Rasch model-data misfit did not necessarily lead to a decreased estimation precision of the sample mean. Rather the effect of model misfit on the RMSE of sample mean seemed to depend on the number of anchor items and was intercepted when the linking was based on at least 5 (20%) anchor items.

### Sample Variance

#### Bias

**FIGURE 3:**
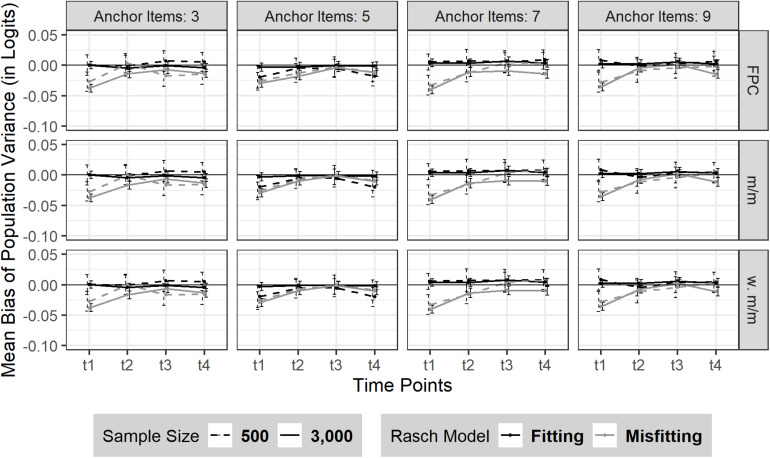
Bias of sample variance over four time points (t_1_–t_4_). The figure is split by three linking methods and the experimental factors number of anchor items, sample size and Rasch model-data fit. FPC = fixed parameter calibration, m/m = mean/mean linking, w. m/m = weighted mean/mean linking. 95% confidence intervals are depicted.

Overall, there was no change in bias or its *SD* over the four time points (*M*_*t1*–*t4*_ = 0.00, *SD*_*t1*–*t4*_ = 0.06) in the absence of model misfit. Neither sample size nor the number of anchor items had a substantial effect on the consistency of the bias of sample variance in the absence of model misfit (see Figure 3). In case of moderate Rasch model-data misfit, the sample variance was marginally underestimated at t_1_ and almost rose back to its true value with measurement progressing. This finding was similarly observed for different number of anchor items and sample size.

#### Relative Bias

The relative bias was considered acceptable in all conditions as it was always below 5% (see Supplementary Table 4).

#### RMSE

**FIGURE 4:**
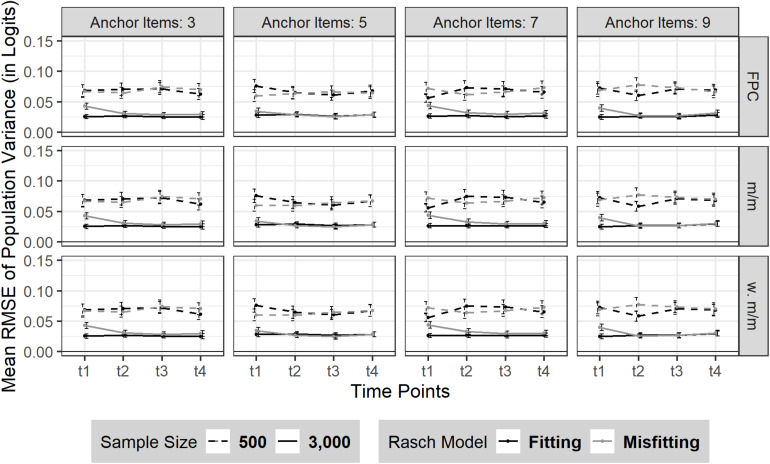
RMSE of sample variance over four time points (t_1_–t_4_). The figure is split by three linking methods and the experimental factors number of anchor items, sample size and Rasch model-data fit. FPC = fixed parameter calibration, Mean/Mean = mean/mean linking, w. Mean/Mean = weighted Mean/Mean. 95% confidence intervals are depicted.

The RMSE of sample variance did not change from t_1_ to t_4_ (see Figure 4). Sample size influenced the amount of RMSE as expected: smaller sample size led to a larger RMSE [*N* = 500: t_1_–t_4_ = 0.07 (*SD*_t1–*t4*_ = 0.05)] compared to a larger sample size [*N* = 3,000: t_1_–t_4_ = 0.03 (*SD*_*t1*–*t4*_ = 0.02)]. No effect was found on the precision of the sample variance estimate due to the number of anchor items or a moderate Rasch model-data misfit.

## Discussion

The present simulation study focused on the comparison of four common IRT-linking methods (fixed parameter calibration, mean/mean linking, weighted mean/mean linking and concurrent calibration) within three experimental conditions (number of anchor items, sample size and model-data fit). Due to convergence issues, the application of concurrent calibration is not advisable for Rasch-scaled data when linking is based on a small absolute number of anchor items. The separate calibration linking methods somewhat unexpectedly resulted in negligible differences in the outcome variables of bias, relative bias and RMSE of sample mean and variance of the latent variable. Hence, the choice of linking method had no effect on the link outcome. This finding may result from the well fitted test targeting at each measurement point in the present study. Thus, even though mean change between time points was substantial (up to 0.7 logits), there were only small differences in measurement precision within each set of anchor items, potentially depriving the method of weighted mean/mean linking of its unique strength in adjusting for differences in anchor item’s *SE*s. Moreover, different amounts of mean change in proficiency over time were handled equally well by the three separate calibration methods. It is to be noted that no differences were found among the three linking methods in sensitivity and reactivity regarding moderate Rasch model-data misfit in the context of longitudinal linking.

In the absence of model misfit, the mean recovery of sample mean and variance was very good, regardless of the sample size or the number of anchor items used. However, in case of moderate Rasch model-data misfit, the parameters of sample mean and variance were generally slightly underestimated, suggesting an influence of the empirical relationship of anchor item difficulty parameters δ*_*i*_* and anchor item discrimination parameters *α_*i*_*. In contrast to prior findings reported in the literature (Zhao and Hambleton, 2017, p. 484), no substantial differences in performance were found between linking methods that based the linking on the anchor item level (e.g., FPC) or the anchor set level (e.g., m/m, wm/m). More specific, a certain composition of δ*_*i*_* and *α_*i*_* in the anchor items seemed to substantially influence the estimation of sample parameters. Factors characterizing this certain composition may include a deviation of item discrimination from 1 on the anchor item and/or anchor set level (i.e., whether misfit is balanced or not), the correlation’s amount and/or direction of δ*_*i*_* and *α_*i*_* as well as person-item fit. Additionally, further investigating the consequences of Rasch model-data misfit seems a promising approach in detangling the compositional effects of anchor items. As the degree of model misfit was assumed to reflect a moderate degree of misfit within the range of operational proficiency test forms, we would furthermore deduce that an increasing degree of model misfit leads to an increasing deviation of parameter estimates from their true parameter.

In the present simulation study, change in proficiency was modeled as decelerating growth in steps of 0.7, 0.5, and 0.3 logits. Nevertheless, the amount of change between two time points seemed independent from the number of anchor items advisable to sufficiently map the change in proficiency distributions of the latent variable. This may suggest a transferability of the present findings to situations in that differences among groups are less pronounced.

It is to be noted, that the consistency of sample mean and variance estimation differed in their sensitivity to the number of anchor items in the case of moderate Rasch model-data misfit. However, accumulating effects (as reported by Keller and Keller, 2011, p. 362–379) of bias were only found when linking was based on 3 (12%) anchor items. While a number of 9 (36%) anchor items seemed sufficient to somewhat balance moderate misfit and resulted in good sample mean recovery, the recovery of sample variance seemed independent of the number of anchor items used. Similarly, for estimation precision of the sample mean, a bigger number of anchor items somewhat attenuated moderate Rasch model-data misfit, although this effect was more beneficial to a smaller sample size. Estimation precision of sample variance seemed to only depend on the sample size.

### Practical Implications

As no substantial impact on parameter recovery of sample mean and variance was found due to moderate Rasch model-data misfit, the Rasch model seemed rather robust in the present context. However, special attention should be payed to anchor items, as their characteristics critically determine sample parameter estimates. Therefore, using a 2PL model seems a practicable diagnostical tool to uncover noticeable deviations in anchor item discrimination parameters. Only marginal differences were found between the three IRT-linking methods of fixed parameter calibration, mean/mean linking and weighted mean/mean linking. More specifically, all of them were equally robust toward a moderate Rasch model-data misfit and different numbers of anchor items even when mean growth was substantial (0.7 logits). As such, the decision for a linking method could rely on more functional factors (e.g., scale preservation, practicability) in case of a well fitted test targeting. If, however, test targeting is expected to be poor, we agree with van der Linden and Barrett (2016, p. 650–673) that weighted mean/mean linking seems to be the preferable choice, as it allows for the inclusion of measurement precision as well as leaving the “pre linking” model fit unaltered. Furthermore, we would like to stress the point that defining an appropriate share of anchor items should depend on the respective Rasch model-data fit rather than following Kolen and Brennan’s (2014) rule of thumb suggesting a share of 20%. In case of moderate misfit, we suggest a number of 7 (36%) anchor items, for the longitudinal linking of short (i.e., 25 items) operational test forms when a Rasch model is used for scaling. Additionally, in case of misfitting anchor items, findings hinted on a compensatory effect when the misfit present is balanced within an anchor item set.

Due to the issues of non-convergence and the disproportionate occurrence of extreme values in parameter recovery, concurrent calibration seemed less suitable for common use than separate calibration methods in longitudinal study designs using small absolute numbers of anchor items.

### Limitations of the Study

The setup of the simulation study did not consider several issues relevant in empirical contexts such as missing data or differential item functioning in anchor items. Similarly, our simulated anchor items exhibited good test targeting for the two proficiency distributions intended to link, which might be hard to achieve in operational assessments. These simplifications of reality were taken into account in order to master the complexity of the central issue. As a consequence, results may be limited in their transferability to empirical data. Future research should study these aspects in more detail and, thus, could further elaborate on the conditions that allow precise linking in the context of the Rasch model. Moreover, the present study was motivated by operational LSAs which are usually characterized by relatively large sample sizes and rather short test forms. In other empirical settings that include smaller sample sizes often substantially longer test forms can be administered. Therefore, future research could address the particulars of linking in these studies. Particularly, this research could also explore whether alternative scaling approaches (e.g., the 2-parameter logistic model) might show more pronounced benefits for data exhibiting misfit to the Rasch model or whether the linking results are comparable to the findings presented in the present study.

As the mean of *α_*i*_* within anchor item sets as well as the correlations of δ*_*i*_* and *α_*i*_* in the present simulation study were not varied systematically, the underlying mechanisms affecting the recovery of sample mean and variance in case of moderate Rasch model-data misfit was not fully traceable and, thus, limited the conclusions on certain compositional effects inherent to sets of anchor items. However, regarding longitudinal measurements, considering the empirical correlation of δ*_*i*_* and *α_*i*_* only, would fall short for the effect of person-item fit. As anchor item difficulties are held constant in repeated administrations to samples with variable proficiencies, person-item fit differs between time points. Therefore, differential effects of an anchor item on the estimation of sample parameters (Bolt et al., 2014, p. 141–162) are to be additionally considered between time points in case of Rasch model-data misfit (Humphry, 2018, p. 216–228).

## Conclusion

Overall, the challenges inherent to contexts characterized by small absolute and relative numbers of anchor items due to short test length as well as small to medium sample sizes were mastered equally well by the three separate calibration methods mean/mean linking, weighted mean/mean linking and fixed parameter calibration, resulting in reliable and valid parameter recovery. However, results of the present simulation study suggested that the choice of linking method is rather secondary when linking Rasch modeled data—independent of the absence or presence of (moderate) model misfit. More important seems the awareness of the practitioner that a combination of moderate model misfit and certain factors (e.g., empirical relation of δ*_*i*_* and *α_*i*_*, composition of anchor items, person-item fit, sample size) may lead to a distorted parameter estimation—although at presence no applicable diagnostics nor concrete guidelines for empirical data seem at hand. As such, future research should analytically deduce and systematically investigate the consequences of an interaction between Rasch model-data misfit and certain experimental factors.

## Data Availability Statement

The original contributions presented in the study are included in the article/Supplementary Material, further inquiries can be directed to the corresponding author/s.

## Author Contributions

LF conducted the literature research, drafted significant parts of the manuscript, and analyzed and interpreted the data used in this study. TG wrote the code for the simulation study. CC, TR, and TG substantively revised the manuscript and provided substantial input for the statistical analyses. All authors read and approved the final manuscript.

## Conflict of Interest

The authors declare that the research was conducted in the absence of any commercial or financial relationships that could be construed as a potential conflict of interest.
