# Supplementary material for: Linking of Rasch-Scaled Tests: Consequences of Limited Item Pools and Model Misfit
Source: Front Psychol. 2021 Jul 6;12:633896. doi: 10.3389/fpsyg.2021.633896 (PMC8289883; doi:10.3389/fpsyg.2021.633896)

Supplementary Material

**Supplementary Table 1.** Convergence Rate of Models Based on Concurrent Calibration Split by Experimental Conditions. Each condition was simulated 100 times. No. of Anchor Items = Number of anchor items; *Md* = Median of the convergence rate of the models based on concurrent calibration split by experimental conditions; Models Converged (%) = model convergence rate in percent.

| Condition | No. of Anchor Items | Rasch Model | Sample Size | Models Converged (%) |
| --- | --- | --- | --- | --- |
| 1 | 3 | Fit | 500 | 21 |
| 2 |  |  | 3,000 | 36 |
| 3 |  | Misfit | 500 | 27 |
| 4 |  |  | 3,000 | 33 |
| 5 | 5 | Fit | 500 | 38 |
| 6 |  |  | 3,000 | 34 |
| 7 |  | Misfit | 500 | 43 |
| 8 |  |  | 3,000 | 44 |
| 9 | 7 | Fit | 500 | 59 |
| 10 |  |  | 3,000 | 51 |
| 11 |  | Misfit | 500 | 64 |
| 12 |  |  | 3,000 | 62 |
| 13 | 9 | Fit | 500 | 74 |
| 14 |  |  | 3,000 | 71 |
| 15 |  | Misfit | 500 | 73 |
| 16 |  |  | 3,000 | 83 |
| *Md* | 30/40.5/60.5/73.5 | 44.5/53 | 51/47.5 | 47.5 |

**Supplementary Table 2.** Descriptive Statistics for the Bias of Sample Mean Split by Experimental Conditions and Linking Methods. Anchor: = Number of anchor items used for linking; *M*(*SD*) = mean and standard deviation of the bias of sample mean; *Min/Max* = *minimum*/*maximum* of the bias of sample mean; *RB* = mean relative bias of sample mean; FPC = fixed parameter calibration; Mean/Mean = mean/mean linking; weighted Mean/Mean = weighted m/m. The bias was averaged over 100 replications.

| **Linking Method** | ***N*** | **Rasch model** | **Time Point** | **Anchor: 3** | | | **Anchor: 5** | | | **Anchor: 7** | | | **Anchor: 9** | | |
| --- | --- | --- | --- | --- | --- | --- | --- | --- | --- | --- | --- | --- | --- | --- | --- |
|  |  |  |  | ***M*(*SD*)** | ***Min*/*Max*** | ***RB*** | ***M*(*SD*)** | ***Min*/*Max*** | ***RB*** | ***M*(*SD*)** | ***Min*/*Max*** | ***RB*** | ***M*(*SD*)** | ***Min*/*Max*** | ***RB*** |
| FPC | 500 | Fit | t_2_ | 0.00(0.09) | -0.25/0.20 | 0.00 | 0.00(0.08) | -0.22/0.26 | 0.00 | -0.01(0.07) | -0.22/0.16 | -0.01 | 0.00(0.06) | -0.14/0.16 | 0.01 |
|  |  |  | t_3_ | -0.01(0.12) | -0.34/0.23 | -0.01 | 0.01(0.12) | -0.28/0.29 | 0.01 | -0.02(0.09) | -0.22/0.19 | -0.01 | 0.01(0.09) | -0.17/0.19 | 0.01 |
|  |  |  | t_4_ | 0.00(0.16) | -0.53/0.46 | 0.00 | 0.01(0.15) | -0.32/0.32 | 0.01 | -0.01(0.10) | -0.24/0.27 | -0.01 | 0.00(0.11) | -0.26/0.19 | 0.00 |
|  |  | Misfit | t_2_ | 0.00(0.09) | -0.18/0.20 | 0.00 | 0.00(0.07) | -0.16/0.22 | -0.01 | -0.03(0.06) | -0.18/0.13 | -0.04 | -0.01(0.06) | -0.13/0.12 | -0.02 |
|  |  |  | t_3_ | -0.02(0.14) | -0.35/0.36 | -0.01 | -0.02(0.11) | -0.38/0.25 | -0.02 | -0.01(0.09) | -0.25/0.31 | -0.01 | -0.01(0.07) | -0.18/0.18 | -0.01 |
|  |  |  | t_4_ | -0.02(0.16) | -0.36/0.34 | -0.01 | -0.03(0.14) | -0.37/0.35 | -0.02 | 0.00(0.11) | -0.32/0.24 | 0.00 | -0.01(0.08) | -0.20/0.19 | 0.00 |
|  | 3,000 | Fit | t_2_ | 0.00(0.04) | -0.12/0.11 | 0.00 | 0.00(0.03) | -0.06/0.06 | 0.00 | 0.00(0.03) | -0.06/0.05 | -0.01 | 0.00(0.02) | -0.06/0.06 | 0.00 |
|  |  |  | t_3_ | 0.00(0.06) | -0.12/0.16 | 0.00 | 0.00(0.04) | -0.09/0.15 | 0.00 | -0.01(0.04) | -0.13/0.09 | 0.00 | 0.00(0.03) | -0.07/0.10 | 0.00 |
|  |  |  | t_4_ | 0.00(0.06) | -0.14/0.17 | 0.00 | 0.00(0.05) | -0.13/0.17 | 0.00 | 0.00(0.05) | -0.12/0.09 | 0.00 | 0.00(0.04) | -0.09/0.11 | 0.00 |
|  |  | Misfit | t_2_ | -0.01(0.04) | -0.10/0.06 | -0.02 | -0.01(0.03) | -0.08/0.06 | -0.01 | -0.04(0.02) | -0.09/0.03 | -0.06 | 0.00(0.02) | -0.07/0.06 | 0.00 |
|  |  |  | t_3_ | -0.05(0.05) | -0.16/0.07 | -0.04 | -0.02(0.04) | -0.11/0.08 | -0.02 | -0.04(0.03) | -0.10/0.05 | -0.03 | 0.00(0.03) | -0.09/0.09 | 0.00 |
|  |  |  | t_4_ | -0.06(0.06) | -0.19/0.09 | -0.04 | -0.01(0.05) | -0.15/0.12 | -0.01 | -0.04(0.04) | -0.12/0.09 | -0.02 | 0.00(0.04) | -0.09/0.11 | 0.00 |
| Mean/Mean | 500 | Fit | t_2_ | 0.00(0.09) | -0.22/0.19 | 0.00 | 0.00(0.08) | -0.23/0.26 | 0.00 | -0.01(0.07) | -0.22/0.15 | -0.01 | 0.00(0.06) | -0.14/0.14 | 0.01 |
|  |  |  | t_3_ | -0.01(0.12) | -0.34/0.22 | 0.00 | 0.01(0.12) | -0.25/0.29 | 0.01 | -0.01(0.09) | -0.23/0.17 | -0.01 | 0.01(0.09) | -0.20/0.20 | 0.01 |
|  |  |  | t_4_ | 0.00(0.16) | -0.53/0.44 | 0.00 | 0.01(0.15) | -0.34/0.32 | 0.01 | -0.01(0.10) | -0.25/0.30 | -0.01 | 0.00(0.11) | -0.28/0.21 | 0.00 |
|  |  | Misfit | t_2_ | 0.01(0.09) | -0.18/0.20 | 0.01 | 0.00(0.07) | -0.15/0.22 | 0.00 | -0.03(0.06) | -0.19/0.14 | -0.04 | -0.01(0.06) | -0.14/0.12 | -0.01 |
|  |  |  | t_3_ | -0.01(0.14) | -0.32/0.38 | -0.01 | -0.01(0.12) | -0.37/0.24 | -0.01 | 0.00(0.09) | -0.23/0.32 | 0.00 | 0.00(0.07) | -0.19/0.20 | 0.00 |
|  |  |  | t_4_ | -0.01(0.16) | -0.33/0.34 | -0.01 | -0.02(0.15) | -0.36/0.35 | -0.01 | 0.01(0.11) | -0.31/0.27 | 0.00 | 0.00(0.09) | -0.20/0.19 | 0.00 |
|  | 3,000 | Fit | t_2_ | 0.00(0.04) | -0.12/0.11 | 0.00 | 0.00(0.03) | -0.06/0.06 | 0.00 | 0.00(0.03) | -0.06/0.05 | -0.01 | 0.00(0.02) | -0.06/0.05 | 0.00 |
|  |  |  | t_3_ | 0.00(0.06) | -0.12/0.16 | 0.00 | 0.00(0.04) | -0.10/0.15 | 0.00 | 0.00(0.04) | -0.11/0.09 | 0.00 | 0.00(0.03) | -0.07/0.10 | 0.00 |
|  |  |  | t_4_ | 0.00(0.06) | -0.15/0.17 | 0.00 | 0.00(0.05) | -0.13/0.16 | 0.00 | 0.00(0.05) | -0.12/0.10 | 0.00 | 0.00(0.04) | -0.08/0.12 | 0.00 |
|  |  | Misfit | t_2_ | -0.01(0.04) | -0.08/0.07 | -0.01 | 0.00(0.03) | -0.07/0.07 | 0.00 | -0.04(0.02) | -0.09/0.03 | -0.06 | 0.00(0.02) | -0.07/0.06 | 0.00 |
|  |  |  | t_3_ | -0.04(0.05) | -0.15/0.08 | -0.03 | -0.01(0.04) | -0.09/0.09 | -0.01 | -0.03(0.03) | -0.10/0.04 | -0.03 | 0.00(0.03) | -0.10/0.09 | 0.00 |
|  |  |  | t_4_ | -0.05(0.06) | -0.18/0.11 | -0.03 | 0.00(0.05) | -0.13/0.13 | 0.00 | -0.03(0.03) | -0.12/0.09 | -0.02 | 0.01(0.04) | -0.07/0.12 | 0.01 |
| weighted Mean/Mean | 500 | Fit | t_2_ | 0.00(0.09) | -0.25/0.17 | -0.01 | 0.00(0.08) | -0.22/0.26 | -0.01 | -0.01(0.07) | -0.23/0.15 | -0.02 | 0.00(0.06) | -0.14/0.16 | 0.00 |
|  |  |  | t_3_ | -0.01(0.11) | -0.33/0.22 | -0.01 | 0.00(0.12) | -0.28/0.28 | 0.00 | -0.02(0.09) | -0.22/0.17 | -0.02 | 0.00(0.09) | -0.18/0.18 | 0.00 |
|  |  |  | t_4_ | -0.01(0.15) | -0.53/0.45 | -0.01 | 0.00(0.15) | -0.33/0.32 | 0.00 | -0.02(0.10) | -0.25/0.26 | -0.01 | -0.01(0.11) | -0.27/0.18 | -0.01 |
|  |  | Misfit | t_2_ | -0.01(0.08) | -0.20/0.19 | -0.02 | -0.01(0.07) | -0.16/0.22 | -0.01 | -0.03(0.06) | -0.18/0.12 | -0.05 | -0.02(0.05) | -0.14/0.11 | -0.03 |
|  |  |  | t_3_ | -0.03(0.14) | -0.36/0.31 | -0.02 | -0.03(0.11) | -0.37/0.24 | -0.02 | -0.02(0.09) | -0.25/0.30 | -0.01 | -0.02(0.07) | -0.19/0.18 | -0.02 |
|  |  |  | t_4_ | -0.03(0.16) | -0.36/0.33 | -0.02 | -0.03(0.14) | -0.37/0.31 | -0.02 | -0.02(0.11) | -0.34/0.24 | -0.01 | -0.02(0.09) | -0.20/0.16 | -0.01 |
|  | 3,000 | Fit | t_2_ | 0.00(0.04) | -0.12/0.11 | 0.00 | 0.00(0.03) | -0.06/0.06 | 0.00 | 0.00(0.03) | -0.07/0.05 | -0.01 | 0.00(0.02) | -0.06/0.06 | 0.00 |
|  |  |  | t_3_ | 0.00(0.06) | -0.12/0.16 | 0.00 | 0.00(0.04) | -0.08/0.15 | 0.00 | -0.01(0.04) | -0.13/0.09 | -0.01 | 0.00(0.03) | -0.07/0.10 | 0.00 |
|  |  |  | t_4_ | 0.00(0.06) | -0.14/0.16 | 0.00 | 0.00(0.05) | -0.14/0.16 | 0.00 | 0.00(0.05) | -0.11/0.10 | 0.00 | 0.00(0.04) | -0.09/0.11 | 0.00 |
|  |  | Misfit | t_2_ | -0.02(0.04) | -0.10/0.05 | -0.03 | 0.00(0.03) | -0.07/0.06 | -0.01 | -0.04(0.02) | -0.10/0.02 | -0.06 | -0.01(0.02) | -0.07/0.06 | -0.01 |
|  |  |  | t_3_ | -0.06(0.05) | -0.17/0.07 | -0.05 | -0.02(0.04) | -0.11/0.09 | -0.01 | -0.04(0.03) | -0.11/0.04 | -0.03 | -0.01(0.03) | -0.10/0.09 | -0.01 |
|  |  |  | t_4_ | -0.07(0.06) | -0.19/0.08 | -0.04 | -0.01(0.05) | -0.16/0.13 | -0.01 | -0.04(0.04) | -0.13/0.08 | -0.03 | 0.00(0.04) | -0.10/0.11 | 0.00 |

**Supplementary Table 3.** Descriptive Statistics for the RMSE of Sample Mean Split by Experimental Conditions and Linking Methods. Anchor: = Number of anchor items used for linking; *M*(*SD*) = mean and standard deviation of the RMSE of sample mean; *Min/Max* = *minimum*/*maximum* of the RMSE of sample mean; FPC = fixed parameter calibration; Mean/Mean = mean/mean linking; weighted Mean/Mean = weighted m/m. The RMSE was averaged over 100 replications.

| **Linking Method** | ***N*** | **Rasch model** | **Time Point** | **Anchor: 3** | | **Anchor: 5** | | | **Anchor: 7** | | **Anchor: 9** | |
| --- | --- | --- | --- | --- | --- | --- | --- | --- | --- | --- | --- | --- |
|  |  |  |  | ***M*(*SD*)** | ***Min*/*Max*** | ***M*(*SD*)** | ***Min*/*Max*** | ***M*(*SD*)** | | ***Min*/*Max*** | ***M*(*SD*)** | ***Min*/*Max*** |
| FPC | 500 | Fit | t_2_ | 0.07(0.05) | 0.00/0.25 | 0.07(0.05) | 0.00/0.26 | 0.05(0.04) | | 0.00/0.22 | 0.05(0.03) | 0.00/0.16 |
|  |  |  | t_3_ | 0.09(0.07) | 0.00/0.34 | 0.10(0.07) | 0.00/0.29 | 0.07(0.05) | | 0.00/0.22 | 0.07(0.05) | 0.00/0.19 |
|  |  |  | t_4_ | 0.12(0.10) | 0.00/0.53 | 0.12(0.09) | 0.00/0.32 | 0.08(0.06) | | 0.00/0.27 | 0.09(0.06) | 0.00/0.26 |
|  |  | Misfit | t_2_ | 0.07(0.05) | 0.00/0.20 | 0.06(0.04) | 0.00/0.22 | 0.05(0.04) | | 0.00/0.18 | 0.05(0.03) | 0.00/0.13 |
|  |  |  | t_3_ | 0.11(0.08) | 0.00/0.36 | 0.09(0.07) | 0.00/0.38 | 0.07(0.06) | | 0.00/0.31 | 0.06(0.04) | 0.00/0.18 |
|  |  |  | t_4_ | 0.13(0.09) | 0.00/0.36 | 0.11(0.09) | 0.00/0.37 | 0.08(0.07) | | 0.00/0.32 | 0.07(0.05) | 0.00/0.20 |
|  | 3,000 | Fit | t_2_ | 0.03(0.03) | 0.00/0.12 | 0.03(0.02) | 0.00/0.06 | 0.02(0.02) | | 0.00/0.06 | 0.02(0.01) | 0.00/0.06 |
|  |  |  | t_3_ | 0.05(0.04) | 0.00/0.16 | 0.03(0.03) | 0.00/0.15 | 0.03(0.02) | | 0.00/0.13 | 0.02(0.02) | 0.00/0.10 |
|  |  |  | t_4_ | 0.05(0.04) | 0.00/0.17 | 0.04(0.04) | 0.00/0.17 | 0.04(0.03) | | 0.00/0.12 | 0.03(0.02) | 0.00/0.11 |
|  |  | Misfit | t_2_ | 0.03(0.02) | 0.00/0.10 | 0.03(0.02) | 0.00/0.08 | 0.04(0.02) | | 0.00/0.09 | 0.02(0.01) | 0.00/0.07 |
|  |  |  | t_3_ | 0.06(0.04) | 0.00/0.16 | 0.04(0.03) | 0.00/0.11 | 0.04(0.03) | | 0.00/0.10 | 0.03(0.02) | 0.00/0.09 |
|  |  |  | t_4_ | 0.07(0.05) | 0.00/0.19 | 0.04(0.03) | 0.00/0.15 | 0.04(0.03) | | 0.00/0.12 | 0.03(0.02) | 0.00/0.11 |
| Mean/Mean | 500 | Fit | t_2_ | 0.07(0.05) | 0.00/0.22 | 0.06(0.05) | 0.00/0.26 | 0.06(0.04) | | 0.00/0.22 | 0.05(0.03) | 0.00/0.14 |
|  |  |  | t_3_ | 0.09(0.07) | 0.00/0.34 | 0.10(0.07) | 0.00/0.29 | 0.08(0.05) | | 0.00/0.23 | 0.07(0.05) | 0.00/0.20 |
|  |  |  | t_4_ | 0.12(0.10) | 0.00/0.53 | 0.12(0.09) | 0.00/0.34 | 0.08(0.06) | | 0.00/0.30 | 0.09(0.06) | 0.00/0.28 |
|  |  | Misfit | t_2_ | 0.07(0.05) | 0.00/0.20 | 0.06(0.04) | 0.00/0.22 | 0.05(0.04) | | 0.00/0.19 | 0.04(0.03) | 0.00/0.14 |
|  |  |  | t_3_ | 0.11(0.08) | 0.00/0.38 | 0.09(0.07) | 0.00/0.37 | 0.07(0.06) | | 0.00/0.32 | 0.06(0.04) | 0.00/0.20 |
|  |  |  | t_4_ | 0.13(0.09) | 0.00/0.34 | 0.12(0.09) | 0.00/0.36 | 0.08(0.07) | | 0.00/0.31 | 0.07(0.05) | 0.00/0.20 |
|  | 3,000 | Fit | t_2_ | 0.03(0.03) | 0.00/0.12 | 0.03(0.02) | 0.00/0.06 | 0.02(0.01) | | 0.00/0.06 | 0.02(0.01) | 0.00/0.06 |
|  |  |  | t_3_ | 0.05(0.04) | 0.00/0.16 | 0.03(0.03) | 0.00/0.15 | 0.03(0.02) | | 0.00/0.11 | 0.02(0.02) | 0.00/0.10 |
|  |  |  | t_4_ | 0.05(0.04) | 0.00/0.17 | 0.04(0.04) | 0.00/0.16 | 0.04(0.03) | | 0.00/0.12 | 0.03(0.02) | 0.00/0.12 |
|  |  | Misfit | t_2_ | 0.03(0.02) | 0.00/0.08 | 0.02(0.02) | 0.00/0.07 | 0.04(0.02) | | 0.00/0.09 | 0.02(0.01) | 0.00/0.07 |
|  |  |  | t_3_ | 0.05(0.04) | 0.00/0.15 | 0.03(0.02) | 0.00/0.09 | 0.04(0.03) | | 0.00/0.10 | 0.03(0.02) | 0.00/0.10 |
|  |  |  | t_4_ | 0.06(0.05) | 0.00/0.18 | 0.04(0.03) | 0.00/0.13 | 0.04(0.03) | | 0.00/0.12 | 0.03(0.02) | 0.00/0.12 |
| weighted Mean/Mean | 500 | Fit | t_2_ | 0.07(0.05) | 0.00/0.25 | 0.06(0.05) | 0.00/0.26 | 0.05(0.04) | | 0.00/0.23 | 0.05(0.03) | 0.00/0.16 |
|  |  |  | t_3_ | 0.09(0.07) | 0.00/0.33 | 0.10(0.07) | 0.00/0.28 | 0.08(0.05) | | 0.00/0.22 | 0.07(0.05) | 0.00/0.18 |
|  |  |  | t_4_ | 0.12(0.09) | 0.00/0.53 | 0.12(0.09) | 0.00/0.33 | 0.08(0.06) | | 0.00/0.26 | 0.09(0.06) | 0.00/0.27 |
|  |  | Misfit | t_2_ | 0.07(0.05) | 0.00/0.20 | 0.06(0.04) | 0.00/0.22 | 0.06(0.04) | | 0.00/0.18 | 0.05(0.03) | 0.00/0.14 |
|  |  |  | t_3_ | 0.11(0.08) | 0.00/0.36 | 0.09(0.07) | 0.00/0.37 | 0.07(0.06) | | 0.00/0.30 | 0.06(0.05) | 0.00/0.19 |
|  |  |  | t_4_ | 0.13(0.09) | 0.00/0.36 | 0.12(0.09) | 0.00/0.37 | 0.08(0.07) | | 0.00/0.34 | 0.07(0.05) | 0.00/0.20 |
|  | 3,000 | Fit | t_2_ | 0.03(0.03) | 0.00/0.12 | 0.03(0.02) | 0.00/0.06 | 0.02(0.01) | | 0.00/0.07 | 0.02(0.01) | 0.00/0.06 |
|  |  |  | t_3_ | 0.05(0.04) | 0.00/0.16 | 0.03(0.03) | 0.00/0.15 | 0.03(0.02) | | 0.00/0.13 | 0.02(0.02) | 0.00/0.10 |
|  |  |  | t_4_ | 0.05(0.04) | 0.00/0.16 | 0.04(0.04) | 0.00/0.16 | 0.04(0.03) | | 0.00/0.11 | 0.03(0.02) | 0.00/0.11 |
|  |  | Misfit | t_2_ | 0.03(0.02) | 0.00/0.10 | 0.02(0.02) | 0.00/0.07 | 0.04(0.02) | | 0.00/0.10 | 0.02(0.02) | 0.00/0.07 |
|  |  |  | t_3_ | 0.06(0.04) | 0.00/0.17 | 0.04(0.02) | 0.00/0.11 | 0.04(0.03) | | 0.00/0.11 | 0.03(0.02) | 0.00/0.10 |
|  |  |  | t_4_ | 0.07(0.05) | 0.00/0.19 | 0.04(0.03) | 0.00/0.16 | 0.04(0.03) | | 0.00/0.13 | 0.03(0.02) | 0.00/0.11 |

**Supplementary Table 4.** Descriptive Statistics for the Bias of Sample Variance Split by Experimental Conditions and Linking Methods. Anchor: = Number of anchor items used for linking; *M*(*SD*) = mean and standard deviation of the bias of sample variance; *Min/Max* = *minimum*/*maximum* of the bias of sample variance; *RB* = mean relative bias of sample variance; FPC = fixed parameter calibration; Mean/Mean = mean/mean linking; weighted Mean/Mean = weighted m/m. The bias was averaged over 100 replications.

| **Linking Method** | ***N*** | **Rasch model** | **Time Point** | **Anchor: 3** | | | **Anchor: 5** | | | **Anchor: 7** | | | **Anchor: 9** | | |
| --- | --- | --- | --- | --- | --- | --- | --- | --- | --- | --- | --- | --- | --- | --- | --- |
|  |  |  |  | ***M*(*SD*)** | ***Min*/*Max*** | ***RB*** | ***M*(*SD*)** | ***Min*/*Max*** | ***RB*** | ***M*(*SD*)** | ***Min*/*Max*** | ***RB*** | ***M*(*SD*)** | ***Min*/*Max*** | ***RB*** |
| FPC | 500 | Fit | t_1_ | 0.00(0.08) | -0.18/0.24 | 0.00 | -0.02(0.09) | -0.18/0.34 | -0.02 | 0.01(0.07) | -0.14/0.16 | 0.01 | 0.01(0.09) | -0.19/0.20 | 0.01 |
|  |  |  | t_2_ | 0.00(0.09) | -0.21/0.23 | 0.00 | 0.00(0.08) | -0.18/0.29 | 0.00 | 0.01(0.09) | -0.20/0.32 | 0.01 | 0.00(0.07) | -0.18/0.18 | 0.00 |
|  |  |  | t_3_ | 0.01(0.09) | -0.21/0.20 | 0.01 | -0.01(0.08) | -0.17/0.19 | -0.01 | 0.01(0.09) | -0.23/0.26 | 0.01 | 0.00(0.09) | -0.18/0.22 | 0.00 |
|  |  |  | t_4_ | 0.01(0.08) | -0.18/0.17 | 0.01 | -0.02(0.08) | -0.25/0.14 | -0.02 | 0.01(0.08) | -0.21/0.28 | 0.01 | 0.01(0.08) | -0.17/0.18 | 0.01 |
|  |  | Misfit | t_1_ | -0.03(0.08) | -0.19/0.20 | -0.03 | -0.03(0.07) | -0.21/0.23 | -0.03 | -0.03(0.08) | -0.17/0.21 | -0.03 | -0.03(0.08) | -0.27/0.23 | -0.03 |
|  |  |  | t_2_ | 0.00(0.08) | -0.17/0.25 | 0.00 | -0.01(0.08) | -0.20/0.18 | -0.01 | -0.01(0.08) | -0.15/0.26 | -0.01 | -0.01(0.10) | -0.21/0.26 | -0.01 |
|  |  |  | t_3_ | -0.02(0.09) | -0.20/0.24 | -0.02 | 0.00(0.08) | -0.19/0.19 | 0.00 | 0.00(0.08) | -0.17/0.19 | 0.00 | 0.00(0.09) | -0.21/0.19 | 0.00 |
|  |  |  | t_4_ | -0.01(0.09) | -0.21/0.19 | -0.01 | -0.01(0.08) | -0.21/0.17 | -0.01 | 0.00(0.09) | -0.24/0.26 | 0.00 | 0.00(0.08) | -0.19/0.23 | 0.00 |
|  | 3,000 | Fit | t_1_ | 0.00(0.03) | -0.07/0.07 | 0.00 | 0.00(0.03) | -0.08/0.07 | 0.00 | 0.00(0.03) | -0.06/0.08 | 0.00 | 0.00(0.03) | -0.10/0.07 | 0.00 |
|  |  |  | t_2_ | 0.00(0.03) | -0.07/0.07 | 0.00 | 0.00(0.04) | -0.10/0.07 | 0.00 | 0.00(0.03) | -0.08/0.09 | 0.00 | 0.00(0.03) | -0.07/0.06 | 0.00 |
|  |  |  | t_3_ | 0.00(0.03) | -0.08/0.07 | 0.00 | 0.00(0.03) | -0.09/0.09 | 0.00 | 0.01(0.03) | -0.06/0.11 | 0.01 | 0.01(0.03) | -0.06/0.07 | 0.01 |
|  |  |  | t_4_ | 0.00(0.03) | -0.10/0.09 | 0.00 | 0.00(0.04) | -0.08/0.09 | 0.00 | 0.00(0.03) | -0.08/0.08 | 0.00 | 0.00(0.03) | -0.08/0.09 | 0.00 |
|  |  | Misfit | t_1_ | -0.04(0.03) | -0.14/0.05 | -0.04 | -0.03(0.03) | -0.11/0.04 | -0.03 | -0.04(0.03) | -0.13/0.04 | -0.04 | -0.04(0.03) | -0.10/0.04 | -0.04 |
|  |  |  | t_2_ | -0.01(0.03) | -0.10/0.06 | -0.01 | -0.02(0.03) | -0.09/0.06 | -0.02 | -0.01(0.04) | -0.12/0.09 | -0.01 | 0.00(0.03) | -0.09/0.06 | 0.00 |
|  |  |  | t_3_ | -0.01(0.03) | -0.09/0.07 | -0.01 | 0.00(0.03) | -0.09/0.10 | 0.00 | -0.01(0.04) | -0.08/0.09 | -0.01 | 0.00(0.03) | -0.10/0.09 | 0.00 |
|  |  |  | t_4_ | -0.01(0.03) | -0.10/0.07 | -0.01 | -0.01(0.03) | -0.10/0.08 | -0.01 | -0.01(0.04) | -0.09/0.09 | -0.01 | -0.01(0.04) | -0.10/0.08 | -0.01 |
| Mean/Mean | 500 | Fit | t_1_ | 0.00(0.08) | -0.18/0.24 | 0.00 | -0.02(0.09) | -0.18/0.34 | -0.02 | 0.01(0.07) | -0.14/0.16 | 0.01 | 0.01(0.09) | -0.19/0.20 | 0.01 |
|  |  |  | t_2_ | 0.00(0.09) | -0.20/0.21 | 0.00 | -0.01(0.08) | -0.19/0.29 | -0.01 | 0.01(0.10) | -0.20/0.31 | 0.01 | 0.00(0.07) | -0.20/0.17 | 0.00 |
|  |  |  | t_3_ | 0.01(0.09) | -0.22/0.21 | 0.01 | -0.01(0.07) | -0.17/0.17 | -0.01 | 0.01(0.09) | -0.21/0.24 | 0.01 | 0.00(0.09) | -0.18/0.23 | 0.00 |
|  |  |  | t_4_ | 0.01(0.08) | -0.18/0.17 | 0.01 | -0.02(0.08) | -0.27/0.15 | -0.02 | 0.01(0.08) | -0.20/0.26 | 0.01 | 0.00(0.08) | -0.18/0.18 | 0.00 |
|  |  | Misfit | t_1_ | -0.03(0.08) | -0.19/0.20 | -0.03 | -0.03(0.07) | -0.21/0.23 | -0.03 | -0.03(0.08) | -0.17/0.21 | -0.03 | -0.03(0.08) | -0.27/0.23 | -0.03 |
|  |  |  | t_2_ | 0.00(0.08) | -0.18/0.25 | 0.00 | -0.01(0.08) | -0.20/0.18 | -0.01 | -0.02(0.08) | -0.16/0.27 | -0.02 | -0.01(0.10) | -0.21/0.27 | -0.01 |
|  |  |  | t_3_ | -0.02(0.09) | -0.21/0.23 | -0.02 | 0.00(0.08) | -0.18/0.18 | 0.00 | 0.00(0.08) | -0.18/0.21 | 0.00 | 0.00(0.09) | -0.24/0.21 | 0.00 |
|  |  |  | t_4_ | -0.02(0.09) | -0.20/0.19 | -0.02 | -0.01(0.08) | -0.21/0.20 | -0.01 | 0.01(0.09) | -0.23/0.28 | 0.01 | 0.00(0.09) | -0.19/0.20 | 0.00 |
|  | 3,000 | Fit | t_1_ | 0.00(0.03) | -0.07/0.07 | 0.00 | 0.00(0.03) | -0.08/0.07 | 0.00 | 0.00(0.03) | -0.06/0.08 | 0.00 | 0.00(0.03) | -0.10/0.07 | 0.00 |
|  |  |  | t_2_ | 0.00(0.03) | -0.07/0.07 | 0.00 | 0.00(0.04) | -0.10/0.07 | 0.00 | 0.00(0.03) | -0.07/0.09 | 0.00 | 0.00(0.03) | -0.07/0.06 | 0.00 |
|  |  |  | t_3_ | 0.00(0.03) | -0.08/0.07 | 0.00 | 0.00(0.03) | -0.10/0.09 | 0.00 | 0.01(0.03) | -0.07/0.11 | 0.01 | 0.01(0.03) | -0.07/0.08 | 0.01 |
|  |  |  | t_4_ | 0.00(0.03) | -0.09/0.10 | 0.00 | 0.00(0.03) | -0.08/0.09 | 0.00 | 0.00(0.03) | -0.08/0.06 | 0.00 | 0.00(0.04) | -0.08/0.10 | 0.00 |
|  |  | Misfit | t_1_ | -0.04(0.03) | -0.14/0.05 | -0.04 | -0.03(0.03) | -0.11/0.04 | -0.03 | -0.04(0.03) | -0.13/0.04 | -0.04 | -0.04(0.03) | -0.10/0.04 | -0.04 |
|  |  |  | t_2_ | -0.02(0.03) | -0.10/0.06 | -0.02 | -0.01(0.03) | -0.08/0.06 | -0.01 | -0.01(0.04) | -0.12/0.08 | -0.01 | -0.01(0.03) | -0.08/0.06 | -0.01 |
|  |  |  | t_3_ | -0.01(0.03) | -0.09/0.07 | -0.01 | 0.00(0.03) | -0.09/0.11 | 0.00 | -0.01(0.04) | -0.08/0.08 | -0.01 | 0.00(0.03) | -0.10/0.09 | 0.00 |
|  |  |  | t_4_ | -0.01(0.04) | -0.10/0.08 | -0.01 | -0.01(0.03) | -0.09/0.08 | -0.01 | -0.01(0.04) | -0.09/0.09 | -0.01 | -0.01(0.04) | -0.10/0.08 | -0.01 |
| weighted Mean/Mean | 500 | Fit | t_1_ | 0.00(0.08) | -0.18/0.24 | 0.00 | -0.02(0.09) | -0.18/0.34 | -0.02 | 0.01(0.07) | -0.14/0.16 | 0.01 | 0.01(0.09) | -0.19/0.20 | 0.01 |
|  |  |  | t_2_ | 0.00(0.09) | -0.20/0.21 | 0.00 | -0.01(0.08) | -0.19/0.29 | -0.01 | 0.01(0.10) | -0.20/0.31 | 0.01 | 0.00(0.07) | -0.20/0.17 | 0.00 |
|  |  |  | t_3_ | 0.01(0.09) | -0.22/0.21 | 0.01 | -0.01(0.07) | -0.17/0.17 | -0.01 | 0.01(0.09) | -0.21/0.24 | 0.01 | 0.00(0.09) | -0.18/0.23 | 0.00 |
|  |  |  | t_4_ | 0.01(0.08) | -0.18/0.17 | 0.01 | -0.02(0.08) | -0.27/0.15 | -0.02 | 0.01(0.08) | -0.20/0.26 | 0.01 | 0.00(0.08) | -0.18/0.18 | 0.00 |
|  |  | Misfit | t_1_ | -0.03(0.08) | -0.19/0.20 | -0.03 | -0.03(0.07) | -0.21/0.23 | -0.03 | -0.03(0.08) | -0.17/0.21 | -0.03 | -0.03(0.08) | -0.27/0.23 | -0.03 |
|  |  |  | t_2_ | 0.00(0.08) | -0.18/0.25 | 0.00 | -0.01(0.08) | -0.20/0.18 | -0.01 | -0.02(0.08) | -0.16/0.27 | -0.02 | -0.01(0.10) | -0.21/0.27 | -0.01 |
|  |  |  | t_3_ | -0.02(0.09) | -0.21/0.23 | -0.02 | 0.00(0.08) | -0.18/0.18 | 0.00 | 0.00(0.08) | -0.18/0.21 | 0.00 | 0.00(0.09) | -0.24/0.21 | 0.00 |
|  |  |  | t_4_ | -0.02(0.09) | -0.20/0.19 | -0.02 | -0.01(0.08) | -0.21/0.20 | -0.01 | 0.01(0.09) | -0.23/0.28 | 0.01 | 0.00(0.09) | -0.19/0.20 | 0.00 |
|  | 3,000 | Fit | t_1_ | 0.00(0.03) | -0.07/0.07 | 0.00 | 0.00(0.03) | -0.08/0.07 | 0.00 | 0.00(0.03) | -0.06/0.08 | 0.00 | 0.00(0.03) | -0.10/0.07 | 0.00 |
|  |  |  | t_2_ | 0.00(0.03) | -0.07/0.07 | 0.00 | 0.00(0.04) | -0.10/0.07 | 0.00 | 0.00(0.03) | -0.07/0.09 | 0.00 | 0.00(0.03) | -0.07/0.06 | 0.00 |
|  |  |  | t_3_ | 0.00(0.03) | -0.08/0.07 | 0.00 | 0.00(0.03) | -0.10/0.09 | 0.00 | 0.01(0.03) | -0.07/0.11 | 0.01 | 0.01(0.03) | -0.07/0.08 | 0.01 |
|  |  |  | t_4_ | 0.00(0.03) | -0.09/0.10 | 0.00 | 0.00(0.03) | -0.08/0.09 | 0.00 | 0.00(0.03) | -0.08/0.06 | 0.00 | 0.00(0.04) | -0.08/0.10 | 0.00 |
|  |  | Misfit | t_1_ | -0.04(0.03) | -0.14/0.05 | -0.04 | -0.03(0.03) | -0.11/0.04 | -0.03 | -0.04(0.03) | -0.13/0.04 | -0.04 | -0.04(0.03) | -0.10/0.04 | -0.04 |
|  |  |  | t_2_ | -0.02(0.03) | -0.10/0.06 | -0.02 | -0.01(0.03) | -0.08/0.06 | -0.01 | -0.01(0.04) | -0.12/0.08 | -0.01 | -0.01(0.03) | -0.08/0.06 | -0.01 |
|  |  |  | t_3_ | -0.01(0.03) | -0.09/0.07 | -0.01 | 0.00(0.03) | -0.09/0.11 | 0.00 | -0.01(0.04) | -0.08/0.08 | -0.01 | 0.00(0.03) | -0.10/0.09 | 0.00 |
|  |  |  | t_4_ | -0.01(0.04) | -0.10/0.08 | -0.01 | -0.01(0.03) | -0.09/0.08 | -0.01 | -0.01(0.04) | -0.09/0.09 | -0.01 | -0.01(0.04) | -0.10/0.08 | -0.01 |

**Supplementary Table 5.** Descriptive Statistics for the RMSE of Sample Variance Split by Experimental Conditions and Linking Methods. Anchor: = Number of anchor items used for linking; *M*(*SD*) = mean and standard deviation of the RMSE of sample variance; *Min/Max* = *minimum*/*maximum* of the RMSE of sample variance; FPC = fixed parameter calibration; Mean/Mean = mean/mean linking; weighted Mean/Mean = weighted m/m. The RMSE was averaged over 100 replications.

| **Linking Method** | ***N*** | **Rasch model** | **Time Point** | **Anchor: 3** | | **Anchor: 5** | | | **Anchor: 7** | | **Anchor: 9** | |
| --- | --- | --- | --- | --- | --- | --- | --- | --- | --- | --- | --- | --- |
|  |  |  |  | ***M*(*SD*)** | ***Min*/*Max*** | ***M*(*SD*)** | ***Min*/*Max*** | ***M*(*SD*)** | | ***Min*/*Max*** | ***M*(*SD*)** | ***Min*/*Max*** |
| FPC | 500 | Fit | t_1_ | 0.07(0.05) | 0.00/0.24 | 0.08(0.06) | 0.00/0.34 | 0.06(0.04) | | 0.00/0.16 | 0.07(0.05) | 0.0/0.20 |
|  |  |  | t_2_ | 0.07(0.05) | 0.00/0.23 | 0.07(0.05) | 0.00/0.29 | 0.07(0.06) | | 0.00/0.32 | 0.06(0.04) | 0.0/0.18 |
|  |  |  | t_3_ | 0.07(0.05) | 0.00/0.21 | 0.06(0.04) | 0.00/0.19 | 0.07(0.06) | | 0.00/0.26 | 0.07(0.05) | 0.0/0.22 |
|  |  |  | t_4_ | 0.06(0.05) | 0.00/0.18 | 0.07(0.05) | 0.00/0.25 | 0.07(0.05) | | 0.00/0.28 | 0.07(0.05) | 0.0/0.18 |
|  |  | Misfit | t_1_ | 0.07(0.05) | 0.00/0.20 | 0.06(0.05) | 0.00/0.23 | 0.07(0.05) | | 0.00/0.21 | 0.07(0.05) | 0.0/0.27 |
|  |  |  | t_2_ | 0.07(0.05) | 0.00/0.25 | 0.06(0.05) | 0.00/0.20 | 0.06(0.05) | | 0.00/0.26 | 0.08(0.06) | 0.0/0.26 |
|  |  |  | t_3_ | 0.07(0.05) | 0.00/0.24 | 0.07(0.05) | 0.00/0.19 | 0.07(0.05) | | 0.00/0.19 | 0.07(0.05) | 0.0/0.21 |
|  |  |  | t_4_ | 0.07(0.05) | 0.00/0.21 | 0.06(0.05) | 0.00/0.21 | 0.07(0.06) | | 0.00/0.26 | 0.07(0.05) | 0.0/0.23 |
|  | 3,000 | Fit | t_1_ | 0.03(0.02) | 0.00/0.07 | 0.03(0.02) | 0.00/0.08 | 0.03(0.02) | | 0.00/0.08 | 0.03(0.02) | 0.0/0.10 |
|  |  |  | t_2_ | 0.03(0.02) | 0.00/0.07 | 0.03(0.02) | 0.00/0.10 | 0.03(0.02) | | 0.00/0.09 | 0.03(0.02) | 0.0/0.07 |
|  |  |  | t_3_ | 0.03(0.02) | 0.00/0.08 | 0.03(0.02) | 0.00/0.09 | 0.03(0.02) | | 0.00/0.11 | 0.03(0.02) | 0.0/0.07 |
|  |  |  | t_4_ | 0.03(0.02) | 0.00/0.10 | 0.03(0.02) | 0.00/0.09 | 0.03(0.02) | | 0.00/0.08 | 0.03(0.02) | 0.0/0.09 |
|  |  | Misfit | t_1_ | 0.04(0.03) | 0.00/0.14 | 0.03(0.03) | 0.00/0.11 | 0.04(0.03) | | 0.00/0.13 | 0.04(0.03) | 0.0/0.10 |
|  |  |  | t_2_ | 0.03(0.02) | 0.00/0.10 | 0.03(0.02) | 0.00/0.09 | 0.03(0.02) | | 0.00/0.12 | 0.03(0.02) | 0.0/0.09 |
|  |  |  | t_3_ | 0.03(0.02) | 0.00/0.09 | 0.03(0.02) | 0.00/0.10 | 0.03(0.02) | | 0.00/0.09 | 0.03(0.02) | 0.0/0.10 |
|  |  |  | t_4_ | 0.03(0.02) | 0.00/0.10 | 0.03(0.02) | 0.00/0.10 | 0.03(0.02) | | 0.00/0.09 | 0.03(0.02) | 0.0/0.10 |
| Mean/Mean | 500 | Fit | t_1_ | 0.07(0.05) | 0.00/0.24 | 0.08(0.06) | 0.00/0.34 | 0.06(0.04) | | 0.00/0.16 | 0.07(0.05) | 0.0/0.20 |
|  |  |  | t_2_ | 0.07(0.05) | 0.00/0.21 | 0.06(0.05) | 0.00/0.29 | 0.08(0.06) | | 0.00/0.31 | 0.06(0.04) | 0.0/0.20 |
|  |  |  | t_3_ | 0.07(0.05) | 0.00/0.22 | 0.06(0.04) | 0.00/0.17 | 0.07(0.06) | | 0.00/0.24 | 0.07(0.05) | 0.0/0.23 |
|  |  |  | t_4_ | 0.06(0.05) | 0.00/0.18 | 0.07(0.05) | 0.00/0.27 | 0.07(0.05) | | 0.00/0.26 | 0.07(0.05) | 0.0/0.18 |
|  |  | Misfit | t_1_ | 0.07(0.05) | 0.00/0.20 | 0.06(0.05) | 0.00/0.23 | 0.07(0.05) | | 0.00/0.21 | 0.07(0.05) | 0.0/0.27 |
|  |  |  | t_2_ | 0.07(0.05) | 0.00/0.25 | 0.06(0.05) | 0.00/0.20 | 0.06(0.05) | | 0.00/0.27 | 0.08(0.06) | 0.0/0.27 |
|  |  |  | t_3_ | 0.07(0.05) | 0.00/0.23 | 0.06(0.05) | 0.00/0.18 | 0.07(0.05) | | 0.00/0.21 | 0.07(0.05) | 0.0/0.24 |
|  |  |  | t_4_ | 0.07(0.05) | 0.00/0.20 | 0.07(0.05) | 0.00/0.21 | 0.07(0.06) | | 0.00/0.28 | 0.07(0.05) | 0.0/0.20 |
|  | 3,000 | Fit | t_1_ | 0.03(0.02) | 0.00/0.07 | 0.03(0.02) | 0.00/0.08 | 0.03(0.02) | | 0.00/0.08 | 0.03(0.02) | 0.0/0.10 |
|  |  |  | t_2_ | 0.03(0.02) | 0.00/0.07 | 0.03(0.02) | 0.00/0.10 | 0.03(0.02) | | 0.00/0.09 | 0.03(0.02) | 0.0/0.07 |
|  |  |  | t_3_ | 0.03(0.02) | 0.00/0.08 | 0.03(0.02) | 0.00/0.10 | 0.03(0.02) | | 0.00/0.11 | 0.03(0.02) | 0.0/0.08 |
|  |  |  | t_4_ | 0.03(0.02) | 0.00/0.10 | 0.03(0.02) | 0.00/0.09 | 0.03(0.02) | | 0.00/0.08 | 0.03(0.02) | 0.0/0.10 |
|  |  | Misfit | t_1_ | 0.04(0.03) | 0.00/0.14 | 0.03(0.03) | 0.00/0.11 | 0.04(0.03) | | 0.00/0.13 | 0.04(0.03) | 0.0/0.10 |
|  |  |  | t_2_ | 0.03(0.02) | 0.00/0.10 | 0.03(0.02) | 0.00/0.08 | 0.03(0.02) | | 0.00/0.12 | 0.03(0.02) | 0.0/0.08 |
|  |  |  | t_3_ | 0.03(0.02) | 0.00/0.09 | 0.02(0.02) | 0.00/0.11 | 0.03(0.02) | | 0.00/0.08 | 0.03(0.02) | 0.0/0.10 |
|  |  |  | t_4_ | 0.03(0.02) | 0.00/0.10 | 0.03(0.02) | 0.00/0.09 | 0.03(0.02) | | 0.00/0.09 | 0.03(0.02) | 0.0/0.10 |
| weighted Mean/Mean | 500 | Fit | t_1_ | 0.07(0.05) | 0.00/0.24 | 0.08(0.06) | 0.00/0.34 | 0.06(0.04) | | 0.00/0.16 | 0.07(0.05) | 0.0/0.20 |
|  |  |  | t_2_ | 0.07(0.05) | 0.00/0.21 | 0.06(0.05) | 0.00/0.29 | 0.08(0.06) | | 0.00/0.31 | 0.06(0.04) | 0.0/0.20 |
|  |  |  | t_3_ | 0.07(0.05) | 0.00/0.22 | 0.06(0.04) | 0.00/0.17 | 0.07(0.06) | | 0.00/0.24 | 0.07(0.05) | 0.0/0.23 |
|  |  |  | t_4_ | 0.06(0.05) | 0.00/0.18 | 0.07(0.05) | 0.00/0.27 | 0.07(0.05) | | 0.00/0.26 | 0.07(0.05) | 0.0/0.18 |
|  |  | Misfit | t_1_ | 0.07(0.05) | 0.00/0.20 | 0.06(0.05) | 0.00/0.23 | 0.07(0.05) | | 0.00/0.21 | 0.07(0.05) | 0.0/0.27 |
|  |  |  | t_2_ | 0.07(0.05) | 0.00/0.25 | 0.06(0.05) | 0.00/0.20 | 0.06(0.05) | | 0.00/0.27 | 0.08(0.06) | 0.0/0.27 |
|  |  |  | t_3_ | 0.07(0.05) | 0.00/0.23 | 0.06(0.05) | 0.00/0.18 | 0.07(0.05) | | 0.00/0.21 | 0.07(0.05) | 0.0/0.24 |
|  |  |  | t_4_ | 0.07(0.05) | 0.00/0.20 | 0.07(0.05) | 0.00/0.21 | 0.07(0.06) | | 0.00/0.28 | 0.07(0.05) | 0.0/0.20 |
|  | 3,000 | Fit | t_1_ | 0.03(0.02) | 0.00/0.07 | 0.03(0.02) | 0.00/0.08 | 0.03(0.02) | | 0.00/0.08 | 0.03(0.02) | 0.0/0.10 |
|  |  |  | t_2_ | 0.03(0.02) | 0.00/0.07 | 0.03(0.02) | 0.00/0.10 | 0.03(0.02) | | 0.00/0.09 | 0.03(0.02) | 0.0/0.07 |
|  |  |  | t_3_ | 0.03(0.02) | 0.00/0.08 | 0.03(0.02) | 0.00/0.10 | 0.03(0.02) | | 0.00/0.11 | 0.03(0.02) | 0.0/0.08 |
|  |  |  | t_4_ | 0.03(0.02) | 0.00/0.10 | 0.03(0.02) | 0.00/0.09 | 0.03(0.02) | | 0.00/0.08 | 0.03(0.02) | 0.0/0.10 |
|  |  | Misfit | t_1_ | 0.04(0.03) | 0.00/0.14 | 0.03(0.03) | 0.00/0.11 | 0.04(0.03) | | 0.00/0.13 | 0.04(0.03) | 0.0/0.10 |
|  |  |  | t_2_ | 0.03(0.02) | 0.00/0.10 | 0.03(0.02) | 0.00/0.08 | 0.03(0.02) | | 0.00/0.12 | 0.03(0.02) | 0.0/0.08 |
|  |  |  | t_3_ | 0.03(0.02) | 0.00/0.09 | 0.02(0.02) | 0.00/0.11 | 0.03(0.02) | | 0.00/0.08 | 0.03(0.02) | 0.0/0.10 |
|  |  |  | t_4_ | 0.03(0.02) | 0.00/0.10 | 0.03(0.02) | 0.00/0.09 | 0.03(0.02) | | 0.00/0.09 | 0.03(0.02) | 0.0/0.10 |

**Supplementary Figure 1.** Anchor-Items Design of the Simulation Study. Four test forms were administered from t_1_ to t_4_, consisting of 25 items each, sharing either a number of 3, 5, 7 or 9 common items with their adjacent test form(s). Blank squares symbolize test form unique items.


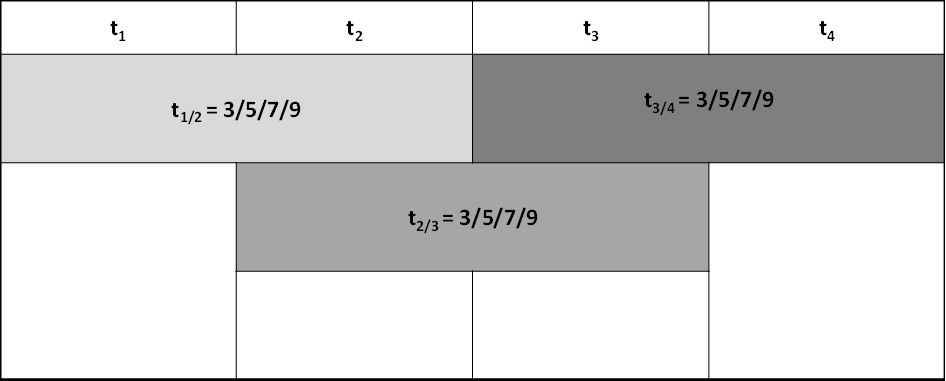

Supplement: Supplementary file 1 [file Data_Sheet_1.docx]
